# Essential Oils from Southern Italian Aromatic Plants Synergize with Antibiotics against *Escherichia coli*, *Pseudomonas aeruginosa* and *Enterococcus faecalis* Cell Growth and Biofilm Formation

**DOI:** 10.3390/antibiotics13070605

**Published:** 2024-06-28

**Authors:** Giada Sena, Elisabetta De Rose, Michele Crudo, Gianfranco Filippelli, Giuseppe Passarino, Dina Bellizzi, Patrizia D’Aquila

**Affiliations:** 1 Department of Biology, Ecology and Earth Sciences, University of Calabria, 87036 Rende, Italy; giada.sena@unical.it (G.S.); elisabetta.derose@unical.it (E.D.R.); giuseppe.passarino@unical.it (G.P.); patrizia.daquila@unical.it (P.D.); 2 Botanical Research Institute of Calabrian Knowledge (B.R.I.C.K.)—GOEL Società Cooperativa Sociale, Via Peppino Brugnano, 89048 Siderno, Italy; michelecrudo22@gmail.com; 3 Unità Operativa Complessa di Oncologia Medica, Ospedale San Francesco di Paola, 87027 Paola, Italy; g.filippelli@tiscali.it

**Keywords:** Essential Oils, antibiotics, *Escherichia coli*, *Pseudomonas aeruginosa*, *Enterococcus faecalis*, MIC, bacterial growth, biofilm, cytosine methylation, adenine methylation

## Abstract

The spread of antibiotic-resistant pathogens has prompted the development of novel approaches to identify molecules that synergize with antibiotics to enhance their efficacy. This study aimed to investigate the effects of ten Essential Oils (EOs) on the activity of nine antibiotics in influencing growth and biofilm formation in *Escherichia coli*, *Pseudomonas aeruginosa*, and *Enterococcus faecalis*. The effects of the EOs alone and in combination with antibiotics on both bacterial growth and biofilm formation were analyzed by measuring the MIC values through the broth microdilution method and the crystal violet assay, respectively. All EOs inhibited the growth of *E. coli* (1.25 ≤ MIC ≤ 5 mg/mL) while the growth of *P. aeruginosa* and *E. faecalis* was only affected by EOs from *Origanum vulgare*, (MIC = 5 mg/mL) and *O. vulgare* (MIC = 1.25 mg/mL) and *Salvia rosmarinus* (MIC = 5 mg/mL), respectively. In *E. coli*, most EOs induced a four- to sixteen-fold reduction in the MIC values of ampicillin, ciprofloxacin, ceftriaxone, gentamicin, and streptomycin, while in *E. faecalis* such a reduction is observed in combinations of ciprofloxacin with *C. nepeta*, *C. bergamia*, *C. limon*, *C. reticulata*, and *F. vulgare*, of gentamicin with *O. vulgare*, and of tetracycline with *C. limon* and *O. vulgare*. A smaller effect was observed in *P. aeruginosa*, in which only *C. bergamia* reduced the concentration of tetracycline four-fold. EO-antibiotic combinations also inhibit the biofilm formation. More precisely, all EOs with ciprofloxacin in *E. coli*, tetracycline in *P. aeruginosa*, and gentamicin in *E. faecalis* showed the highest percentage of inhibition. Combinations induce up- and down-methylation of cytosines and adenines compared to EO or antibiotics alone. The study provides evidence about the role of EOs in enhancing the action of antibiotics by influencing key processes involved in resistance mechanisms such as biofilm formation and epigenetic changes. Synergistic interactions should be effectively considered in dealing with pathogenic microorganisms.

## 1. Introduction

Bacterial infections, which were dramatically reduced by the introduction of antibiotics, currently represent a major health problem due to growing bacterial resistance and related increases in care costs and mortality rates. In Europe, antibiotic resistance exhibited by several pathogens causes more than 33,000 deaths every year, and about one-third of these occur in Italy, which is one of the European countries with the highest percentages of resistant microorganisms as well as the biggest consumer of antibiotics [1]. The problem is even more serious if we consider that strains of bacteria resistant not only to a single drug but to many are spreading both in the community and hospitals due to the improper use of antibiotics. Hence the need, in recent years, to develop different approaches to identify new molecules that can represent alternative agents to antibiotics. As regards this, a few studies have demonstrated that Essential Oils (EOs), odorous and volatile products from the secondary metabolism of aromatic plants, can represent a distinctive group of possible novel drugs effective in interfering with the growth and pathogenicity-associated biological processes of a wide range of microorganisms [2]. During the last few years, numerous EOs have been identified, which could have important therapeutic potential due to their high diversity of chemical compounds such as terpenoids (monoterpene, sesquiterpene, and diterpene) and phenylpropane derivatives [3,4,5,6,7]. Among these EOs, those extracted from *Verbenaceae* (*Verbena officinalis*), *Apiaceae* (*Foeniculum vulgare*, *Carum carvi*), *Lamiaceae* (*Origanum vulgare*, *Clinopodium nepeta*, *Rosmarinus officinalis*, *Salvia officinalis*, *Salvia rosmarinus*, *Majorana hortensis*, *Thymus vulgaris*, *Lavandula stoechas*, *Menta piperita*), *Lauraceae* (*Laurus nobilis*), *Myrtaceae* (*Myrtus communis*, *Pimenta dioica*), and *Rutaceae* (*Citrus clementina*, *Citrus bergamia*, *Citrus limon*, *Citrus reticulata*, *Murraya koenigii*) have been reported not only to have anti-growth properties but also to suppress Quorum Sensing (QS) and biofilm formation, two well-known processes that confer multidrug resistance and influence the expression of bacterial virulence factors in pathogenic microorganisms such as *Staphylococcus aureus*, *Escherichia coli*, *Enterococcus faecalis*, *Klebsiella pneumoniae*, and *Pseudomonas aeruginosa* [8,9,10,11,12]. In recent years, various approaches have been developed, including the combination of antibiotics with EOs, to investigate the synergic effect of them, which could be more effective in counteracting bacterial resistance [13,14,15,16,17]. Romo-Castillo et al. demonstrated the potential of EOs extracted from *Rosmarinus officinalis*, *Thymus vulgaris*, *Mentha piperita*, and *Curcuma longa* to combat *K. pneumoniae* by applying a synergic therapy for antibiotics [18]. The effects of *Protium heptaphyllum* EO (PHEO) alone and in combination with antibiotics against polymyxin-resistant *K. pneumoniae* isolates were also analyzed [19]. A synergistic interaction was observed for EOs from *Foeniculum vulgare* and *Lavandula angustifolia* with amoxicillin against *S. aureus*, *E. faecalis*, *Bacillus cereus*, *E. coli*, and *Salmonella typhi* [20]. The combination of *Citrus microcarpa* oil and tetracycline exhibited a synergistic effect on growth and biofilm formation [21]. It was reported that the EO from *Origanum vulgare* exerts antibacterial activity and enhances the action of antibiotics against extended-spectrum β-lactamase-producing *E. coli* [22]. 

In this study, we aimed to investigate whether ten EOs, which we have previously shown to have antibacterial activity [8,11], improve the efficacy of nine commercial antibiotics on growth and biofilm formation in *E. coli*, *P. aeruginosa*, and *E. faecalis* cells. *E. coli* is a Gram-negative bacterium frequently used as a model organism, while *P. aeruginosa* and *E. faecalis* are, respectively, Gram-negative and Gram-positive common opportunistic pathogens that are frequently identified in biofilm infections and cause both severe acute and chronic infections, often associated with healthcare.

## 2. Results

### 2.1. Antimicrobial Activity of the Essential Oils

Ten EOs belonging to Apiaceae (*Foeniculum vulgare* subsp. Piperitum), *Lamiaceae* (*Clinopodium nepeta*, *Origanum vulgare* L. subsp. *Viridulum*, *Salvia officinalis* L., *Salvia rosmarinus*), *Lauraceae* (*Laurus nobilis* L.), *Myrtaceae* (*Myrtus communis* L.) and *Rutaceae* (*Citrus bergamia*, *Citrus limon*, *Citrus reticulata*) family plants were analyzed in the present study. A complete description of all EO components and their percentages, and a Retention Index, is reported in Table S1. The analysis revealed that (+)-limonene represents the predominant component of EOs from *C. bergamia* and *C. limon*, while eucalyptol is the major detected compound in EOs from *L. nobilis*, *M. communis*, *S. officinalis*, and *S. rosmarinus*. EOs from *C. nepeta*, *C. reticulata*, *F. vulgare*, and *O. vulgare* were characterized by high levels of piperitone oxide, (+)-sabinene, estragole, and p-thymol, respectively. 

Under our laboratory conditions, the antibacterial activity of the EOs against the Gram-negative *E. coli* and *P. aeruginosa*, and the Gram-positive *E. faecalis* was evaluated by determining the MIC values which are shown in Table 1. 

All EOs had strong antimicrobial effects against *E. coli*. In particular, the EO from *O. vulgare* showed the greatest inhibitory effect (1.25 mg/mL), followed by *C. bergamia*, *F. vulgare*, and *S. rosmarinus* (2.5 mg/mL). All EOs tested in *P. aeruginosa* exhibited MIC values greater than 5 mg/mL, except for EO extracted from *O. vulgare*, which showed an MIC value of 5 mg/mL. As regards *E. faecalis*, the most effective EO was *O. vulgare* (1.25 mg/mL). We also observed a MIC value around 5 mg/mL for *S. rosmarinus* meanwhile the remaining EOs displayed MIC values greater than 5 mg/mL. 

In Table 1, the MICs of ampicillin (AMP), aztreonam (AZT), ciprofloxacin (CIP), ceftriaxone (CTR), erythromycin (ERY), gentamicin (GEN), meropenem (MER), streptomycin (STR), and tetracycline (TET) are also reported. 

The MIC values of EOs and antibiotics were used to calculate the FIC_I_ in evaluating the effects against bacteria when they were analyzed in combination with each other. 

### 2.2. Effects on the MIC Values of Antibiotics Induced by Their Combination with EOs

The significant effects of combinations of AMP, AZT, CIP, CTR, ERY, GEN, MER, STR, and TET with EOs against *E. coli*, *P. aeruginosa*, and *E. faecalis* are reported in Table 2, as MIC gain of antibiotics.

In *E. coli*, the gain we observed in the MIC values of AMP were four-fold (*C. reticulata* and *O. vulgare*) and two-fold (*L. nobilis* and *S. rosmarinus*); of CIP, eight-fold (*C. nepeta*, *C. limon*, *C. reticulata*, *F. vulgare*, *M. communis*, *S. officinalis*, and *S. rosmarinus*), four-fold (*O. vulgare*), and two-fold (*C. bergamia* and *L. nobilis*); CTR, eight-fold (*C. bergamia*, *C. reticulata*, *L. nobilis*, *O. vulgare*, and *S. rosmarinus*) and four-fold (*C. limon* and *F. vulgare*); ERY, two-fold (*C. nepeta*, *C. bergamia*, *C. reticulata*, *O. vulgare*, *S. officinalis*, and *S. rosmarinus*); GEN, eight-fold (*C. reticulata*), four-fold (*F. vulgare* and *O. vulgare*), and two-fold (*C. nepeta*, *C. bergamia*, *C. limon*, *M. communis*, *S. officinalis*, and *S. rosmarinus*); and, lastly, of STR, sixteen-fold (*C. reticulata* and *O. vulgare*), eight-fold (*C. bergamia*, *F.* vulgare, *S. officinalis*, and *S. rosmarinus*), and four-fold (*C. nepeta*, *C. limon*, and *M. communis*). 

In *P. aeruginosa*, we observed a two-fold gain in the MIC values of AMP (*C. nepeta*, *C. bergamia*, *C. limon*, *L. nobilis*, *O. vulgare*, *S. officinalis*, and *S. rosmarinus*), AZT (*C. nepeta*, *C. bergamia*, *C. limon*, *C. reticulata*, *M. communis*, *O. vulgare*, *S. officinalis*, and *S. Rosmarinus*), CIP (*C. nepeta*, *C. limon*, *F. vulgare*, *L. nobilis*, *M. communis*, and *O. vulgare*), CTR (*O. vulgare*), GEN (*C. bergamia*, *C. limon*, *F. vulgare*, *M. communis*, *O. vulgare*, and *S. rosmarinus*), MER (*C. reticulata* and *S. Rosmarinus*), and TET (*C. nepeta*, *C. limon*, *C. reticulata*, *F. vulgare*, *S. officinalis*, and *S. rosmarinus*). A four-fold gain against these organisms was obtained for C. bergamia in combination with TET. 

Lastly, in *E. faecalis*, we observed a two-fold gain in the MIC values of AMP (all EOs except for *C. bergamia*), CIP (*L. nobilis*, *M. communis*, *O. vulgare*, *S. officinalis*, and *S. rosmarinus*), CTR (*C. reticulata*, *F. vulgare*, *L. nobilis*, *M. communis*, *O. vulgare*, *S. officinalis*, and *S. rosmarinus*), ERY (*O. vulgare*), GEN (*C. nepeta*, *F. vulgare*, *L. nobilis*, *M. communis*, and *S. rosmarinus*), STR (*C. nepeta*, *F. vulgare*, and *S. rosmarinus*), and TET (*F. vulgare* and *S. rosmarinus*). More pronounced results, with a sixteen-fold gain, were noted in the MICs of both CIP and TET combined with *C. limon*; an eight-fold gain in the MIC of CIP with *C. nepeta*; and a four-fold gain in the MICs of CIP (*C. bergamia*, *C. reticulata*, *F. vulgare*), GEN (*O. vulgare*), and TET (*O. vulgare*). A schematic representation of the changes induced in the MIC values of antibiotics by their combinations with EOs is reported in Figure S1.

The FIC_I_ of the combinations of EOs with antibiotics is shown in Table 3.

Among the 90 combinations, in *E. coli*, 26 showed synergism and 19 showed additivity; in *P. aeruginosa*, 9 showed synergism and 30 showed additivity; and in *E. faecalis*, 9 showed synergism and 29 showed additivity. No interaction was observed for the remaining combinations and none of the combinations showed antagonistic effects in the three microorganisms.

### 2.3. Effects on Biofilm Formation Induced by Combinations of EOs with Antibiotics

**Figure 1:**
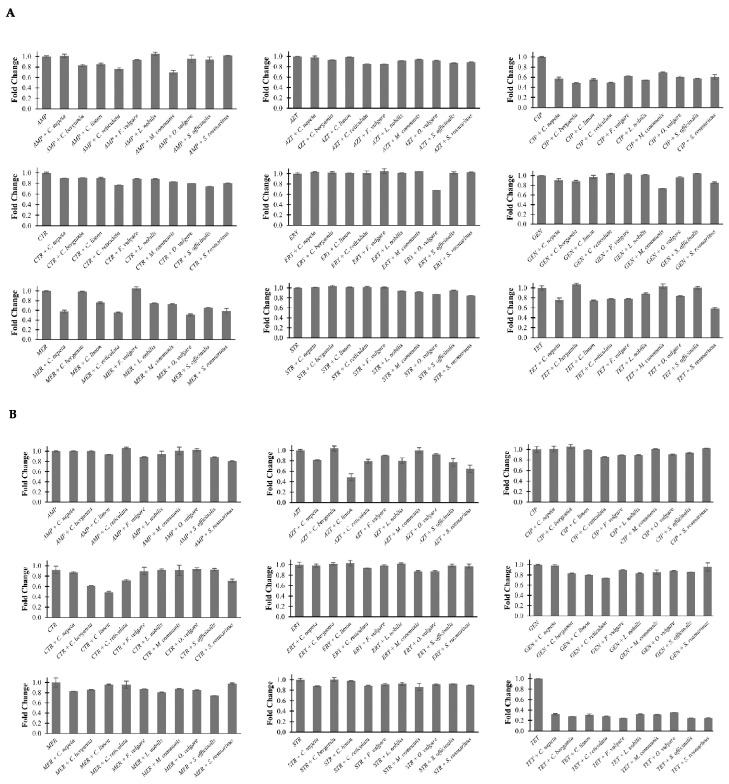

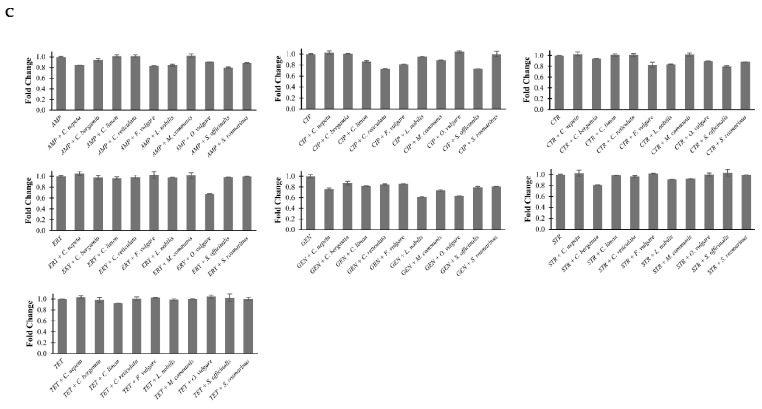
Effects induced by the combination of EOs and antibiotics on biofilm formation in *E. coli* (**A**), *P. aeruginosa* (**B**), and *E. faecalis* (**C**). Values are reported in terms of fold change determined using cells treated with the antibiotic alone as reference. Values represent the average of three independent experiments with a standard error of the mean. AMP: ampicillin, AZT: aztreonam, CIP: ciprofloxacin, CTR: ceftriaxone, ERY: erythromycin, GEN: gentamicin, MER: meropenem, STR: streptomycin, TET: tetracycline.

The biofilm formation activity of the three strains was evaluated for the EOs in combination with each antibiotic through the crystal violet dye assay. Figure 1 shows the statistically significant effects of these combinations, in which they are reported in terms of fold change compared to the activity of the antibiotic alone. Most combinations of the EOs with antibiotics produce a substantial inhibition of bacterial biofilm formation. In *E. coli*, we noted inhibition ranging from 35% to 20% for combinations of *C. bergamia*, *C. limon*, *C. reticulata*, and *M. communis* with AMP; from 13% to 7% for combinations of *C. bergamia*, *C. reticulata*, *F. vulgare*, *M. communis*, *O. vulgare*, *S. officinalis*, and *S. rosmarinus* with AZT; from 52% to 30% for combinations of all EOs analyzed with CIP; from 26% to 10% for combinations of all EOs analyzed with CTR; from 27% to 10% for combinations of *C. nepeta*, *C. bergamia*, *M. communis*, and *S. rosmarinus* for combinations with GEN; from 50% to 25% for combinations of *C. nepeta*, *C. limon*, *C. reticulata*, *L. nobilis*, *M. communis*, *O. vulgare*, *S. officinalis*, and *S. rosmarinus* with MER; from 16% to 6% for combinations of *L. nobilis*, *M. communis*, *O. vulgare*, *S. officinalis*, and *S. rosmarinus* with STR; and from 42% to 12% for combinations of *C. nepeta*, *C. limon*, *C. reticulata*, *F. vulgare*, *L. nobilis*, *O. vulgare*, and *S. rosmarinus* with TET. As regards ERY, the sole combination inducing a significant reduction of biofilm activity was with *O. vulgare* (Figure 1A).

In *P. aeruginosa*, we observed inhibition ranging from 20% to 7% for combinations of *C. limon*, *O. vulgare*, *S. officinalis*, and *S. rosmarinus* with AMP; from 51% to 8% for combinations of *C. nepeta*, *C. limon*, *C. reticulata*, *F. vulgare*, *L. nobilis*, *O. vulgare*, *S. officinalis*, and *S. rosmarinus* with AZT; from 15% to 10% for combinations of *C. reticulata*, *F. vulgare*, *L. nobilis*, *O. vulgare* with CIP; from 51% to 29% for combinations of *C. bergamia*, *C. limon*, *C. reticulata*, and *S. rosmarinus* with CTR; from 13% to 7% for combinations of *C. reticulata*, *M. communis*, and *O. vulgare* with ERY; from 27% to 11% for combinations of *C. bergamia*, *C. limon*, *C. reticulata*, *F. vulgare*, *L. nobilis*, *M. communis*, *O. vulgare*, and *S. officinalis* with GEN; from 26% to 13% for combinations of *C. nepeta*, *C. bergamia*, *F. vulgare*, *L. nobilis*, *M. communis*, *O. vulgare*, and *S. officinalis* with MER; from 13% to 10% for combinations of *C. nepeta*, *C. reticulata*, *L. nobilis*, *O. vulgare*, *S. officinalis*, and *S. rosmarinus* with STR; and from 75% to 65% for combinations of the EOs with TET (Figure 1B).

In *E. faecalis*, we observed inhibition ranging from 20% to 6% for combinations of *C. nepeta*, *C. bergamia*, *F. vulgare*, *L. nobilis*, *O. vulgare*, *S. officinalis*, and *S. rosmarinus* with AMP; from 27% to 5% for combinations of *C. limon*, *C. reticulata*, *F. vulgare*, *L. nobilis*, *M. communis*, and *S. officinalis* with CIP; from 20% to 6% for combinations of *C. bergamia*, *F. vulgare*, *L. nobilis*, *O. vulgare*, *S. officinalis*, and *S. rosmarinus* with CTR; from 39% to 13% for combinations of all EOs analyzed with GEN; and from 19% to 8% for combinations of *C. bergamia*, *L. nobilis*, *M. communis* with STR. As regards ERY and TET, the only combinations that induced inhibition of biofilm formation were with *O. vulgare* (33%) and *C. limon* (8%), respectively (Figure 1C). For all other combinations, we did not observe any significant differences.

### 2.4. Effects of EOs in Combination with Antibiotics on Bacterial Methylation Profiles

**Figure 2:**
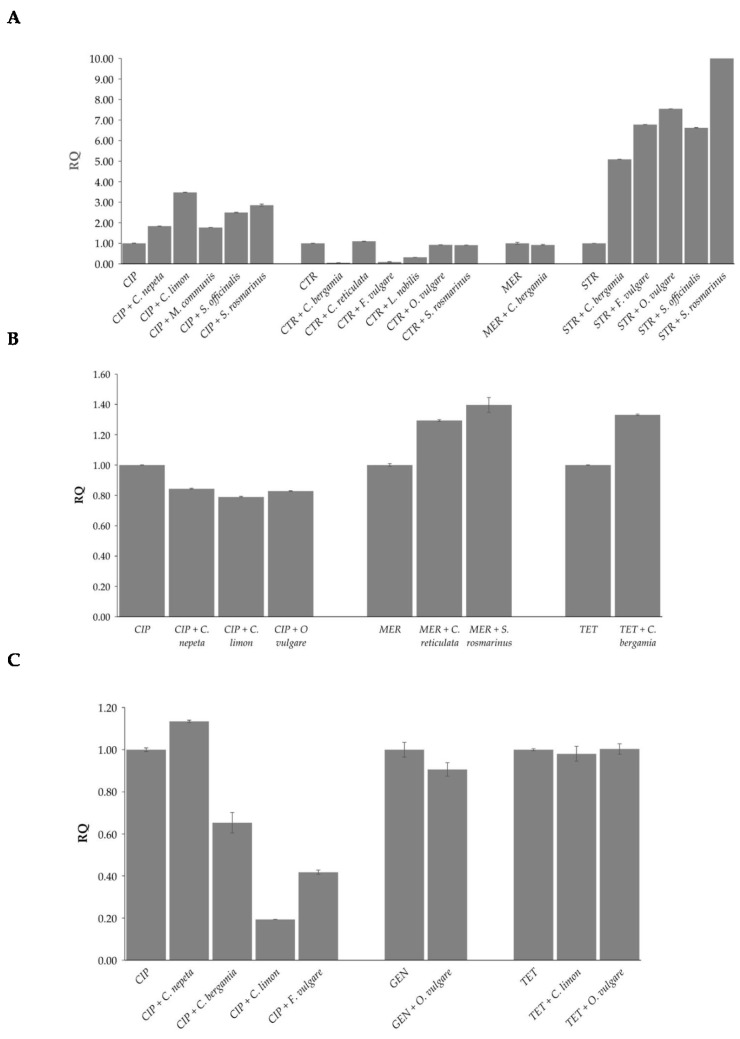
Effects induced by the combinations of EOs and antibiotics on methylation of adenines in *E. coli* (**A**), *P. aeruginosa* (**B**), and *E. faecalis* (**C**). Values are reported as Relative Quantification (RQ) determined using cells treated with the antibiotic alone as reference. Values represent the average of three independent experiments with a standard error of the mean. CIP: ciprofloxacin, CTR: ceftriaxone, GEN: gentamicin, MER: meropenem, STR: streptomycin, TET: tetracycline.

**Figure 3:**
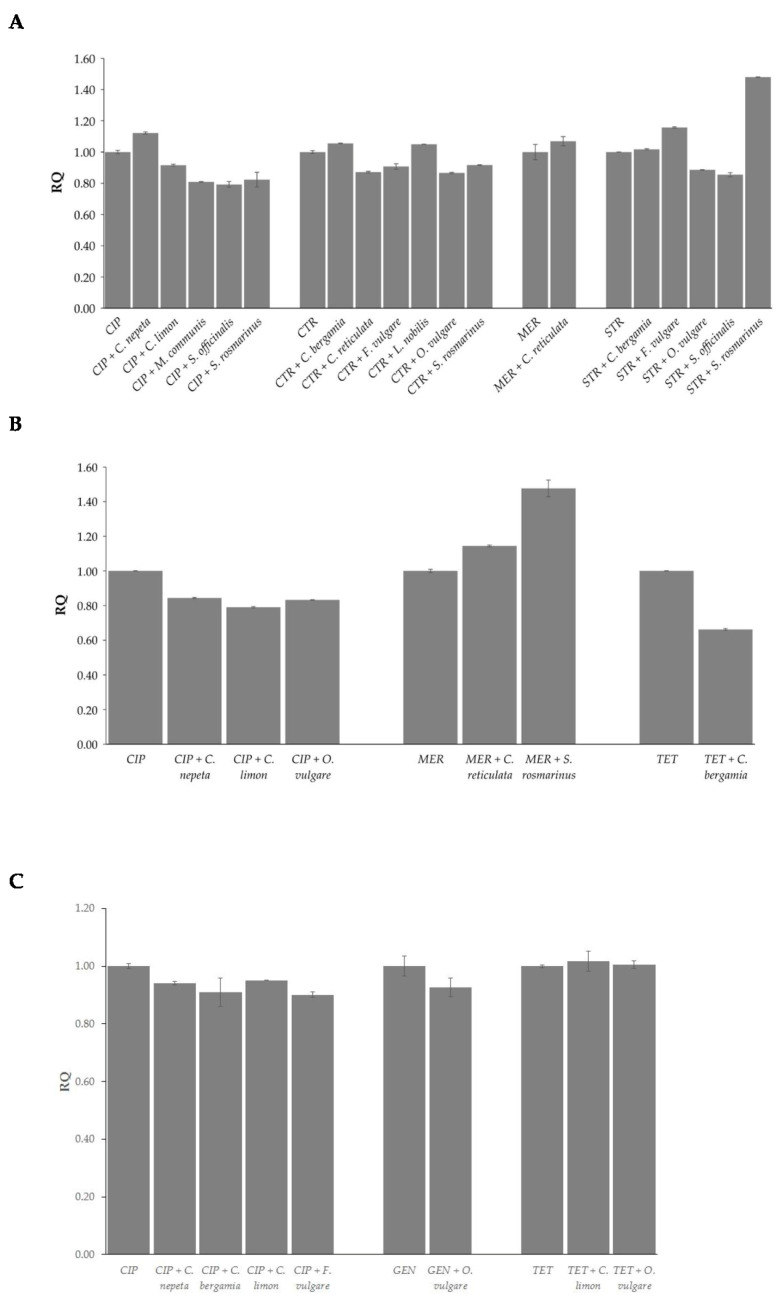
Effects induced by the combinations of EOs and antibiotic on methylation of cytosines in *E. coli* (**A**), *P. aeruginosa* (**B**), and *E. faecalis* (**C**). Values are reported as Relative Quantification (RQ) determined using cells treated with the antibiotic alone as reference. Values represent the average of three independent experiments with a standard error of the mean. CIP: ciprofloxacin, CTR: ceftriaxone, GEN: gentamicin, MER: meropenem, STR: streptomycin, TET: tetracycline.

The global methylation levels of adenine and cytosine residues were evaluated in DNA samples extracted from the three strains at sub-MIC concentrations where synergistic effects of the combinations of EOs and antibiotics were observed. By comparing global DNA methylation levels of both adenine (m6A) and cytosine (5mC) following the combination of EOs and antibiotics with those of the antibiotics alone, statistically significant differences were observed according to antibiotic, EO, and strain (Figure 2 and Figure 3).

As concerns m6A levels, in *E. coli*, all EOs induced up-methylation in combination with ciprofloxacin and streptomycin, while *C. bergamia*, *F. vulgare*, and *L. nobilis* induced down-methylation in combination with ceftriaxone. No statistically significant changes were observed for *C. reticulata*, *O. vulgare*, and *S. rosmarinus* combinations with ceftriaxone or for *C. bergamia* with meropenem (Figure 2A). In *P. aeruginosa*, analyzed EOs induced up-methylation in combination with meropenem and tetracycline and down-methylation in combination with ciprofloxacin (Figure 2B). In *E. faecalis*, *C. nepeta* induced up-methylation and *C. bergamia*, *C. limon*, and *F. vulgare* induced down-methylation in combination with ciprofloxacin. No statistically significant changes were observed for *O. vulgare* in combination with gentamicin or for *C. limon* and *O. vulgare* in combination with tetracycline (Figure 2C).

As concerns 5mC levels, in *E. coli*, *C. nepeta* induced up-methylation while *C. limon*, *M. communis*, *S. officinalis*, and *S. rosmarinus* induced down-methylation in combination with ciprofloxacin; *C. bergamia*, and *L. nobilis* induced up-methylation while *C. reticulata*, *F. vulgare*, *O. vulgare*, and *S. rosmarinus* induced down-methylation in combination with ceftriaxone; *F. vulgare*, and *S. rosmarinus* induced up-methylation while *O. vulgare* and *S. officinalis* induced down-methylation in combination with streptomycin. No statistically significant changes were observed for *C. reticulata* in combination with meropenem or for *C. bergamia* with streptomycin (Figure 3A). In *P. aeruginosa*, *C. reticulata*, and *S. rosmarinus* induced up-methylation in association with meropenem, while a reduction in methylation levels emerged from the combinations of *C. nepeta*, *C. limon*, and *O. vulgare* with ciprofloxacin and *C. bergamia* with tetracycline (Figure 3B). In *E. faecalis*, a decrease in methylation levels was observed following combination of *C. nepeta*, *C. bergamia*, *C. limon*, and *F. vulgare* with ciprofloxacin. No statistically significant changes were observed for *O. vulgare* in combination with gentamicin or for *C. limon* and *O. vulgare* in combination with tetracycline (Figure 3C).

## 3. Discussion

In two previous studies, we reported that EOs extracted from *C. nepeta*, *C. bergamia*, *C. limon*, *C. reticulata*, *F. vulgare*, *L. nobilis*, *M. communis*, *O. vulgare*, *S. officinalis*, and *S. rosmarinus* aromatic plants showed antibacterial activity against *E. coli*, *Chromobacterium violaceum*, and *E. faecalis* [8,11]. Furthermore, in the last two strains, the antibiofilm properties of the EOs were also demonstrated. These results have increased the amount of evidence that is accumulating about the biological role of EOs, especially considering their possible application in the treatment of bacterial infections [23,24,25,26,27]. 

In the present study, we wanted to explore combinations of the ten EOs with commercial antibiotics, which belong to different classes and have different mechanisms of action, to identify the ones with synergistic effects and which, therefore, reduce the minimum dose necessary for each drug to exert its antibacterial effect. The identification of these combinations could suggest the adoption of new therapeutic approaches to be used not exclusively against pathogenic bacteria but mainly in combating multi-resistant bacteria, which are progressively increasing and against which classic antibiotics seem to no longer be effective [17,28,29]. 

Among the EOs analyzed, EO from *O. vulgare* exhibited the highest antimicrobial activity in both *E. coli* and *E. faecalis* (MIC = 1.25 mg/mL), confirming the results of two of our previous studies [8,11]. This result is also in line with literature data in which the antimicrobial efficacy of this EO was demonstrated by the low MIC values determined not only with respect to the above bacteria but also with others such as *S. aureus*, *Salmonella enteritidis*, *Salmonella Typhimurium, Listeria monocytogenes*, and *Bacillus subtilis* [30,31,32,33,34,35,36]. Interestingly, this EO is the only one that, in the conditions we adopted, inhibits bacterial growth in *P. aeruginosa*. Man et al., in a comparative study carried out on a group of pathogens, including *E. coli*, *P. aeruginosa*, and *E. faecalis*, reported that the only EO that presented bactericidal effects on all tested bacteria was precisely that extracted from *O. vulgare* [37]. The remarkable efficiency of the EO could be ascribed to the presence of components such as thymol, terpinene, and p-cymene, which were present at the highest percentages in the oil we analyzed. An extensive description of the modes of action of these components, to fully understand how they might exert their effects on bacterial growth, could refer to two reviews in which it was reported that the common mechanism of action in EO from *O. vulgare* involves suppression of enterotoxin production, release of cellular content, permeabilization of membranes, leakage of potassium and phosphate, dissipation of pH gradients, depletion of motive proton force, and coagulation of the cytoplasm [17,31]. However, the precise molecular mechanisms of the individual bioactive molecules that constitute the EOs we analyzed will require specific and extensive future investigation.

As regards the other EOs in our study, they showed antibacterial activity in *E. coli* with MIC values ranging between 2.5 and 5 mg/mL, while in *E. faecalis* only *S. rosmarinus* showed antibacterial activity, with a MIC of 5 mg/mL. The variability between these MIC values with those we have reported in our previous work may be due to differences in the type of EO solvent adopted (DMSO versus inulin) and the system adopted (microdilution versus macrodilution broth method). The heterogeneity that emerges from literature data is primarily attributable to the different chemical characteristics of EOs, which derives from the cultivar, altitude, temperature, harvest season, geographical position, and extraction method. In addition, the genetic diversity of bacterial strains and the culture media may be responsible for different sensitivities to agents with antibacterial activity [38,39,40]. Regardless of all these variability factors, what clearly emerges is that bacterial growth is susceptible to the action of EOs at low concentrations in non-pathogenic organisms such as *E. coli*, while higher concentrations are required in pathogenic organisms such as *E. faecalis* and even more so in *P. aeruginosa*.

The combinations of EOs with most of the drugs we analyzed (ampicillin, aztreonam, ceftriaxone, ciprofloxacin, erythromycin, gentamicin, meropenem, streptomycin, and tetracycline) appeared particularly effective in reducing the concentration of antibiotics needed to inhibit bacterial growth, as most EOs at least halved it in the three bacterial strains. The combination has shown greater effectiveness in *E. coli*, in which 4-, 8- and 16-fold MIC gains were achieved with ampicillin, ciprofloxacin, ceftriaxone, gentamicin, and streptomycin in association with most of the EOs tested. In *E. faecalis*, a similar reduction in MIC values was reached for ciprofloxacin in combination with *C. nepeta*, *C. bergamia*, *C. limon*, *C. reticulata*, and *F. vulgare*; for gentamicin in combination with *O. vulgare*; and in tetracycline in combination with *C. limon* and *O. vulgare*. A smaller effect was observed in *P. aeruginosa*, in which only *C. bergamia* reduced the concentration of tetracycline four-fold. Therefore, these results represent further confirmation of the series of data in the literature which have highlighted the ability of EOs to enhance the effects of antibiotics in oil-, antibiotic-, and strain-specific manner. Similarly to those we analyzed, EOs containing carvacrol, eucalyptol, eugenol, and thymol have been demonstrated to exercise a synergistic effect in combination with antibiotics [17,18,28,41]. Findings observed in *P. aeruginosa* appear relevant if we consider that it is widely recognized as a prototype of a “multidrug-resistant pathogen” that is frequently associated with increased morbidity and mortality. Indeed, although the oils are by themselves unable to significantly counteract the growth of this pathogen, their use in association with specific antibiotics appears significantly effective by minimizing the dose of drug and, therefore, significantly reducing the risk of selection of resistant bacteria. 

The ability to form biofilms is one of the most effective strategies that bacterial cells implement to ensure survival in adverse conditions. To our knowledge, this is the first study that has simultaneously analyzed combinations of several EOs with multiple antibiotics against biofilm formation in the three microorganisms, leading to results similar to those reported in literature [42,43]. The association of all the EOs with ciprofloxacin in *E. coli*, tetracycline in *P. aeruginosa*, and gentamicin in *E. faecalis* gave the best results in terms of biofilm inhibition. Likely, the lipophilic nature of the EOs decreases bacterial hydrophobicity, thereby decreasing adhesive ability and interfering with bacterial biofilm formation [42]. Also, with regard to biofilm inhibition, the variability we observed suggests that specific interactions are established between each EO and antibiotic that differentially influence the above process. 

Mechanisms which may contribute to synergy between EOs and antibiotics have been reported by various authors and include the involvement of efflux or afflux pumps, alterations in membrane permeability, and modification of substrate, although the association of antibiotics with EOs may involve alternative mechanisms of action not yet characterized [44,45,46,47]. Among these, the results we obtained are extremely interesting because they reveal, for the first time, that the effects of combinations of EOs and antibiotics can be mediated by epigenetic modifications of the bacterial genomes, thus shedding light on the molecular mechanisms through which these combinations act at the intracellular level. Our analyses have painted a very complex landscape of methylation profiles since, in most cases, we observed both up- and down-methylation in both cytosine and adenine residues after treatment with EOs combined with antibiotics, depending on EOs, antibiotics, and strains. As it has not yet been well defined whether DNA methylation in bacteria is mainly associated with gene silencing, the precise effects of these epigenetic changes on gene expression will be the subject of future studies. 

## 4. Materials and Methods

### 4.1. Bacterial Strains and Growth Conditions

This study was carried out on *E. coli* strain JM109 (Stratagene, La Jolla, CA, USA), *P. aeruginosa* (ATCC-9027), and *E. faecalis* OG1RF (ATCC 47077). *E. coli* cells were cultured in Luria-Bertani medium containing 10 g of Bacto-Tryptone, 5 g of yeast extract, and 0.5 g of sodium chloride. *P. aeruginosa* were cultured in nutrient broth containing 3 g/L of beef extract and 5 g/L of peptone at 30 °C. Meanwhile, *E. faecalis* was cultured in 36.4 g/L of Todd–Hewitt broth (THB) (Oxoid, Basingstoke, Hampshire, UK) at 37 °C under gentle agitation. The bacterial strains were kept frozen in stock cultures at −80 °C in cryovials.

### 4.2. EOs and Antibiotics

Ten EOs were obtained from a commercial producer located in Calabria (Briatico (VV) and Bovalino (RC), Italy). They were extracted from the following plants: *Clinopodium nepeta* L. (Kuntze, Carl Ernst Otto), *Citrus bergamia* (Risso, Joseph Antoine & Poiteau, Pierre Antoine), *Citrus limon* L. (Osbeck, Pehr), *Citrus reticulata* (Blanco, Francisco Manuel), *Foeniculum vulgare* subsp. *piperitum*, *Laurus nobilis* L., *Myrtus communis* L., *Origanum vulgare* L. subsp. *viridulum* (Martrin-Donos, Julien Victor) Nyman, Carl Frederik, *Salvia officinalis* L., and *Salvia rosmarinus* (Spenn, Fridolin Carl). Their extraction procedure and composition analysis through GC–MS were described in D’Aquila et al. (2023) [8]. Since EOs are highly lipophilic organic mixtures, we dissolved them in 2% DMSO in growth medium to obtain a working solution of 10 mg EO/mL. The percentage of DMSO was selected after testing a wide series of concentrations of DMSO to identify the one that did not impact on bacterial growth.

Ampicillin (AMP), aztreonam (AZT), ciprofloxacin (CIP), ceftriaxone (CTR), erythromycin (ERY), gentamicin (GEN), meropenem (MER), streptomycin (STR), and tetracycline (TET) (Sigma-Aldrich, St Louis, MO, USA) were analyzed in the study. Working solutions of all antibiotics were prepared at a final concentration of 5000 μg/mL.

### 4.3. Determination of Minimum Inhibitory Concentration (MIC) of the EOs and Antibiotics

The Minimum Inhibitory Concentration (MIC) of the EOs and antibiotics was determined by the broth microdilution method carried out in multi-well microplate [48,49]. Approximately 10^4^ cells of *E. coli*, *P. aeruginosa*, and *E. faecalis* from overnight cultures were inoculated into 96-well plates containing 1:2 serial dilutions of the working solution of EOs or antibiotics at final volume of 150 μL. Microplates were incubated for 22 h at 37 °C. In all experiments, medium with (positive control) and without (negative control) cells and free of EOs and antibiotics were also analyzed to check the adequacy of microbial growth and sterility, respectively. In addition, two further controls were represented by a cell-free medium in the presence of each EO to discern the turbidity background, as well as by medium-containing cells in the presence of 2% DMSO free of EOs, to confirm in each experiment the absence of any effect on bacterial growth. Turbidity measurement was performed at 600 nm in a spectrophotometer. MIC values were determined as the lowest concentrations of Essential Oils and antibiotics corresponding to values of optical density (OD) comparable to those of the cell-free medium. Each experiment was carried out in triplicate, with three independent repetitions.

### 4.4. Synergy between Essential Oils and Antibiotics Evaluation

The effects of EOs on the activity of the antibiotics was assessed. Approximately 10^4^ cells of *E. coli*, *P. aeruginosa*, and *E. faecalis* from overnight cultures were inoculated into the wells containing 1:2 serial dilutions of the working solution of EOs and dilutions of antibiotics ranging from five steps below the MIC to concentrations twice the MIC (Table S2). To test the type of interaction between EOs and antibiotics, the Fractional Inhibitor Concentration Index (FIC_I_) was calculated using the following formula: FIC_I_ = FIC_EO_ + FIC _antibiotic_, with FIC_EO_ = (MIC of EO in combination with antibiotic)/(MIC of EO alone), and FIC _antibiotic_ = (MIC of antibiotic in combination with EO)/(MIC of antibiotic alone). The results were interpreted as total synergism when FIC_I_ ≤ 0.5; 0.5 < FIC_I_ ≤ 1 indicates additivity; 1 < FICI ≤ 4 no interaction; and FICI > 4 antagonistic [50,51]. The MIC gain of the antibiotics was calculated according to the following formula: MIC gain = MIC of antibiotic alone/MIC of antibiotic in combination.

### 4.5. Biofilm Formation Assay

Approximately 10^4^ cells of *E. coli*, *P. aeruginosa*, and *E. faecalis* from an overnight culture were inoculated into 96-well polystyrene microtiter plates containing 150 µL of growth medium in the absence and presence of dilutions of EOs and antibiotics as described in the previous paragraph [52]. After 18 h of incubation, the planktonic cells were removed, and adherent cells were fixed with methanol and then stained with crystal violet solution 0.2%. After 5 min, the excess of the stain was removed by three repeated washes. Acetic acid at a concentration of 33% was added to each well and the optical density at 570 nm was determined spectrophotometrically. Each strain was examined in duplicate on each plate and the experiments were performed in quintuplicate. Positive and negative controls containing only the inoculated growth medium and the growth medium supplemented with EOs, respectively, were run in each experiment.

### 4.6. DNA Extraction

Genomic DNA was extracted from cell growth in the presence of combinations of EOs and antibiotics at sub-MIC concentrations, where a synergistic effect was observed. A DNeasy UltraClean Microbial Kit (Qiagen, Milan, Italy) was used according to the manufacturer’s protocol. Briefly, 3 mL pellets of bacterial cell culture were suspended in 300 µL of PowerBead solution and vortexed. Resuspended cells were transferred to PowerBead tubes and then 50 µL of Solution SL was added. After vortexing for 10 min, the tubes were centrifuged at 10,000× *g* for 30 s. A total of 100 µL of Solution IRS was added to the supernatants and incubated at 4 °C for 5 min. After centrifugation at 10,000× *g* for 1 min, 900 µL of Solution SB was added to the supernatants. Subsequently, 700 µL of sample was loaded into MB Spin Columns and centrifuged at 10,000× *g* for 30 s. The centrifugation was repeated after adding 300 µL of Solution CB and the flow-through discarded. DNA samples were eluted by centrifugation at 10,000× *g* for 30 s in 50 µL of Solution EB. Samples of cell growth in the absence of EOs and antibiotics and samples maintained in the presence of only growth medium and of combinations of EOs and antibiotics in the absence of cells (negative control) were subjected to the same procedure. The DNA concentration and purity were determined spectrophotometrically, and the purity of the sample evaluated using the 260/280 nm absorbance ratio.

### 4.7. Quantification of Global N6-Methyladenosine and 5-Methylcytosine Levels

Global DNA methylation levels of N6-methyladenosines (m6A) and 5-methylcytosines (5mC) were determined by using the MethylFlash m6A DNA Methylation ELISA Kit (Epigentek, Farmingdale, Nassau County, NY, USA) and the MethylFlash Global DNA Methylation (5mC) ELISA Easy Kit, respectively, following the manufacturer’s instructions. Shortly, the methylated fraction of bacterial genomic DNA, through ELISA-like reactions, was recognized by the m6A or 5mC antibodies and quantified in a microplate spectrophotometer by reading the absorbance at 450 nm. In each experiment, the percentage of m6A and 5mC was calculated using the second-order regression equation of a standard curve that was constructed by mixing equivalent molar concentrations at different ratios of full unmethylated and methylated control DNA. Each sample was analyzed in triplicate. The methylation values of DNA samples extracted from cells treated with antibiotics alone were used as reference values (relative quantification, RQ) for the corresponding samples treated with combinations of EOs and antibiotics. 

### 4.8. Statistical Analysis

Statistical analyses were performed using SPSS 28.0 statistical software (SPSS Inc., Chicago, IL, USA). Kruskal–Wallis one-way analysis of variance and Mann-Whitney tests were adopted. Significance level was defined as *p* ≤ 0.05.

## 5. Conclusions

Results obtained corroborate the antibacterial activity of the EOs and provide evidence that they could be used in association with traditional antibiotic therapy to minimize the effective dose of traditional drugs. These associations are effective not only in influencing bacterial growth but also in processes fundamental to the development of antibiotic resistance, such as biofilm formation. With the escalating increase of multidrug-resistant bacteria, developing a therapeutic approach consisting of EOs that synergistically act with antibiotics to counteract microbial proliferation and reduce the risk of selection of resistant bacteria appears fundamental. The use of EOs probably will not totally inhibit antibiotic resistance, but it could contribute to reduce antibiotic use.

## Figures and Tables

**Table 1:** MIC values of the Essential Oils (mg/mL) and the antibiotics (µg/mL) examined in the study.

		*E. coli*	*P. aeruginosa*	*E. faecalis*
Essential Oils	*C. nepeta*	5	>5	>5
	*C. bergamia*	2.5	>5	>5
	*C. limon*	5	>5	>5
	*C. reticulata*	5	>5	>5
	*F. vulgare*	2.5	>5	>5
	*L. nobilis*	5	>5	>5
	*M. communis*	5	>5	>5
	*O. vulgare*	1.25	5	1.25
	*S. officinalis*	5	>5	>5
	*S. rosmarinus*	2.5	>5	5
Antibiotics	AMP	50	1000	5
	AZT	0.09	20	>4000
	CIP	0.6	0.20	4
	CTR	0.15	50	6.25
	ERY	300	315	500
	GEN	12.5	0.80	25
	MER	0.05	0.06	50
	STR	12.5	2	250
	TET	5	200	3

AMP: ampicillin, AZT: aztreonam, CIP: ciprofloxacin, CTR: ceftriaxone, ERY: erythromycin, GEN: gentamicin, MER: meropenem, STR: streptomycin, TET: tetracycline.

**Table 2:** Gain in MIC values of ampicillin (AMP), aztreonam (AZT), ciprofloxacin (CIP), ceftriaxone (CTR), erythromycin (ERY), gentamicin (GEN), meropenem (MER), streptomycin (STR), and tetracycline (TET) in combination with the EOs against *E. coli*, *P. aeruginosa*, and *E. faecalis*.

*E. coli*			*P. aeruginosa*			*E. faecalis*		
AMP	*C. reticulata*	4	AMP	*C. nepeta*	2	AMP	*C. nepeta*	2
	*L. nobilis*	2		*C. bergamia*	2		*C. limon*	2
	*O. vulgare*	4		*C. limon*	2		*C. reticulata*	2
	*S. rosmarinus*	2		*L. nobilis*	2		*F. vulgare*	2
CIP	*C. nepeta*	8		*O. vulgare*	2		*L. nobilis*	2
	*C. bergamia*	2		*S. officinalis*	2		*M. communis*	2
	*C. limon*	8		*S. rosmarinus*	2		*O. vulgare*	2
	*C. reticulata*	8	AZT	*C. nepeta*	2		*S. officinalis*	2
	*F. vulgare*	8		*C. bergamia*	2		*S. rosmarinus*	2
	*L. nobilis*	2		*C. limon*	2	CIP	*C. nepeta*	8
	*M. communis*	8		*C. reticulata*	2		*C. bergamia*	4
	*O. vulgare*	4		*M. communis*	2		*C. limon*	16
	*S. officinalis*	8		*O. vulgare*	2		*C. reticulata*	4
	*S. rosmarinus*	8		*S. officinalis*	2		*F. vulgare*	4
CTR	*C. bergamia*	8		*S. rosmarinus*	2		*L. nobilis*	2
	*C. limon*	4	CIP	*C. nepeta*	2		*M. communis*	2
	*C. reticulata*	8		*C. limon*	2		*O. vulgare*	2
	*F. vulgare*	4		*F. vulgare*	2		*S. officinalis*	2
	*L. nobilis*	8		*L. nobilis*	2		*S. rosmarinus*	2
	*O. vulgare*	8		*M. communis*	2	CTR	*C. reticulata*	2
	*S. rosmarinus*	8		*O. vulgare*	2		*F. vulgare*	2
ERY	*C. nepeta*	2	CTR	*O. vulgare*	2		*L. nobilis*	2
	*C. bergamia*	2	GEN	*C. bergamia*	2		*M. communis*	2
	*C. reticulata*	2		*C. limon*	2		*O. vulgare*	2
	*O. vulgare*	2		*F. vulgare*	2		*S. officinalis*	2
	*S. officinalis*	2		*M. communis*	2		*S. rosmarinus*	2
	*S. rosmarinus*	2		*O. vulgare*	2	ERY	*O. vulgare*	2
GEN	*C. nepeta*	2		*S. rosmarinus*	2	GEN	*C. nepeta*	2
	*C. bergamia*	2	MER	*C. reticulata*	2		*F. vulgare*	2
	*C. limon*	2		*S. rosmarinus*	2		*L. nobilis*	2
	*C. reticulata*	8	TET	*C. nepeta*	2		*M. communis*	2
	*F. vulgare*	4		*C. bergamia*	4		*O. vulgare*	4
	*M. communis*	2		*C. limon*	2		*S. rosmarinus*	2
	*O. vulgare*	4		*C. reticulata*	2	STR	*C. nepeta*	2
	*S. officinalis*	2		*F. vulgare*	2		*F. vulgare*	2
	*S. rosmarinus*	2		*S. officinalis*	2		*S. rosmarinus*	2
STR	*C. nepeta*	4		*S. rosmarinus*	2	TET	*C. limon*	16
	*C. bergamia*	8					*F. vulgare*	2
	*C. limon*	4					*O. vulgare*	4
	*C. reticulata*	16					*S. rosmarinus*	2
	*F. vulgare*	8						
	*M. communis*	4						
	*O. vulgare*	16						
	*S. officinalis*	8						
	*S. rosmarinus*	8						

**Table 3:** FIC_I_ of ampicillin (AMP), aztreonam (AZT), ciprofloxacin (CIP), ceftriaxone (CTR), erythromycin (ERY), gentamicin (GEN), meropenem (MER), streptomycin (STR), and tetracycline (TET) in combination with the EOs against *E. coli*, *P. aeruginosa*, and *E. faecalis*.

		*E. coli*				*P. aeruginosa*				*E. faecalis*	
		FIC_I_	Effect			FIC_I_	Effect			FIC_I_	Effect
AMP	*C. reticulata*	0.75	Additive	AMP	*C. nepeta*	0.6	Additive	AMP	*C. nepeta*	0.92	Additive
	*L. nobilis*	0.63	Additive		*C. bergamia*	0.6	Additive		*C. limon*	0.92	Additive
	*O. vulgare*	0.75	Additive		*C. limon*	0.6	Additive		*C. reticulata*	0.92	Additive
	*S. rosmarinus*	1	Additive		*L. nobilis*	0.6	Additive		*F. vulgare*	0.92	Additive
CIP	*C. nepeta*	0.25	Synergistic		*O. vulgare*	0.6	Additive		*L. nobilis*	0.92	Additive
	*C. limon*	0.25	Synergistic		*S. officinalis*	0.6	Additive		*M. communis*	0.92	Additive
	*C. reticulata*	0.25	Synergistic		*S. rosmarinus*	0.6	Additive		*S. officinalis*	0.92	Additive
	*F. vulgare*	0.38	Synergistic	AZT	*C. nepeta*	0.69	Additive		*S. rosmarinus*	1	Additive
	*L. nobilis*	1	Additive		*C. bergamia*	0.69	Additive	CIP	*C. nepeta*	0.23	Synergistic
	*M. communis*	0.25	Synergistic		*C. limon*	0.69	Additive		*C. bergamia*	0.44	Synergistic
	*S. officinalis*	0.25	Synergistic		*C. reticulata*	0.69	Additive		*C. limon*	0.11	Synergistic
	*S. rosmarinus*	0.38	Synergistic		*M. communis*	0.69	Additive		*C. reticulata*	0.44	Synergistic
CTR	*C. nepeta*	0.33	Synergistic		*O. vulgare*	0.69	Additive		*F. vulgare*	0.44	Synergistic
	*C. bergamia*	0.07	Synergistic		*S. officinalis*	0.69	Additive		*L. nobilis*	0.92	Additive
	*C. limon*	0.09	Synergistic		*S. rosmarinus*	0.69	Additive		*M. communis*	0.92	Additive
	*C. reticulata*	0.04	Synergistic	CIP	*C. nepeta*	0.31	Synergistic		*S. officinalis*	0.92	Additive
	*F. vulgare*	0.15	Synergistic		*C. bergamia*	0.61	Additive		*S. rosmarinus*	1	Additive
	*L. nobilis*	0.04	Synergistic		*C. limon*	0.31	Synergistic	CTR	*C. limon*	0.92	Additive
	*M. communis*	0.33	Synergistic		*C. reticulata*	0.61	Additive		*C. reticulata*	0.92	Additive
	*O. vulgare*	0.14	Synergistic		*F. vulgare*	0.31	Synergistic		*F. vulgare*	0.92	Additive
	*S. officinalis*	0.33	Synergistic		*L. nobilis*	0.31	Synergistic		*L. nobilis*	0.92	Additive
	*S. rosmarinus*	0.07	Synergistic		*M. communis*	0.31	Synergistic		*M. communis*	0.92	Additive
ERY	*C. nepeta*	0.73	Additive		*O. vulgare*	0.31	Synergistic		*S. rosmarinus*	1	Additive
	*C. bergamia*	0.95	Additive		*S. officinalis*	0.61	Additive	ERY	*O. vulgare*	0.6	Additive
	*C. reticulata*	0.73	Additive		*S. rosmarinus*	0.61	Additive	GEN	*C. nepeta*	0.92	Additive
	*S. officinalis*	0.73	Additive	GEN	*C. bergamia*	0.71	Additive		*F. vulgare*	0.92	Additive
	*S. rosmarinus*	0.95	Additive		*C. limon*	0.71	Additive		*L. nobilis*	0.92	Additive
GEN	*C. nepeta*	0.75	Additive		*F. vulgare*	0.71	Additive		*M. communis*	0.92	Additive
	*C. bergamia*	1	Additive		*M. communis*	0.71	Additive		*O. vulgare*	0.44	Synergistic
	*C. limon*	0.75	Additive		*O. vulgare*	0.71	Additive		*S. rosmarinus*	0.92	Additive
	*C. reticulata*	0.19	Synergistic		*S. rosmarinus*	0.71	Additive	STR	*C. nepeta*	0.57	Additive
	*F. vulgare*	0.5	Synergistic	MER	*C. reticulata*	0.5	Synergistic		*F. vulgare*	0.57	Additive
	*M. communis*	0.75	Additive		*S. rosmarinus*	0.5	Synergistic		*S. rosmarinus*	0.5	Synergistic
	*O. vulgare*	0.75	Additive	TET	*C. nepeta*	0.92	Additive	TET	*C. limon*	0.09	Synergistic
	*S. officinalis*	0.75	Additive		*C. bergamia*	0.46	Synergistic		*F. vulgare*	0.69	Additive
	*S. rosmarinus*	1	Additive		*C. limon*	0.92	Additive		*O. vulgare*	0.35	Synergistic
STR	*C. nepeta*	0.88	Additive		*C. reticulata*	0.92	Additive		*S. rosmarinus*	0.69	Additive
	*C. bergamia*	0.37	Synergistic		*F. vulgare*	0.92	Additive				
	*C. limon*	0.5	Synergistic		*S. officinalis*	0.92	Additive				
	*C. reticulata*	0.12	Synergistic		*S. rosmarinus*	0.92	Additive				
	*F. vulgare*	0.37	Synergistic								
	*M. communis*	0.88	Additive								
	*O. vulgare*	0.31	Synergistic								
	*S. officinalis*	0.25	Synergistic								
	*S. rosmarinus*	0.37	Synergistic								

## Data Availability

Data are contained within the article and Supplementary Materials.

## Supplementary Materials

The following supporting information can be downloaded at: https://www.mdpi.com/article/10.3390/antibiotics13070605/s1, Figure S1. Schematic representation of the changes induced in the MIC values of antibiotics by their combinations with EOs. Table S1. Chemical components of the Essential Oils analysed in the study (presence ≥ 1%). Table S2. Scalar concentrations of each antibiotic (mg/mL) tested in combination with the scalar dilutions of EOs (mg/mL) for *E. coli*, *P. aeruginosa* and *E. faecalis*.
