# Supplementary material for: Essential Oils from Southern Italian Aromatic Plants Synergize with Antibiotics against Escherichia coli, Pseudomonas aeruginosa and Enterococcus faecalis Cell Growth and Biofilm Formation
Source: Antibiotics (Basel). 2024 Jun 28;13(7):605. doi: 10.3390/antibiotics13070605 (PMC11274178; doi:10.3390/antibiotics13070605)
Supplement: Supplementary file 1 [file antibiotics-13-00605-s001.zip › antibiotics-3056142-supplementary.pdf]

		Ampicillin	Aztreonam	Ciprofloxacin	Ceftriaxone	Erytromycin	Gentamicin	Meropenem	Streptomycin	Tetracycline
E. coli	*C. nepeta*	▬	▬	↓	▬	↓	↓	▬	↓	▬
	C. bergamia	▬	▬	↓	↓	↓	↓	▬	↓	▬
	C. limon	▬	▬	↓	↓	▬	↓	▬	↓	▬
	C. reticulata	↓	▬	↓	↓	↓	↓	▬	↓	▬
	F. vulgare	▬	▬	↓	↓	▬	↓	▬	↓	▬
	L. nobilis	↓	▬	↓	↓	▬	▬	▬	▬	▬
	M. communis	▬	▬	↓	▬	▬	↓	▬	↓	▬
	O. vulgare	↓	▬	↓	↓	↓	↓	▬	↓	▬
	S. officinalis	▬	▬	↓	▬	↓	↓	▬	↓	▬
	S. rosmarinus	↓	▬	↓	↓	↓	↓	▬	↓	▬

		Ampicillin	Aztreonam	Ciprofloxacin	Ceftriaxone	Erytromycin	Gentamicin	Meropenem	Streptomycin	Tetracycline
P. aeruginosa	*C. nepeta*	↓	↓	↓	▬	▬	▬	▬	▬	↓
	C. bergamia	↓	↓	▬	▬	▬	↓	▬	▬	↓
	C. limon	↓	↓	↓	▬	▬	↓	▬	▬	↓
	C. reticulata	▬	↓	▬	▬	▬	▬	↓	▬	↓
	F. vulgare	▬	▬	↓	▬	▬	↓	▬	▬	↓
	L. nobilis	↓	▬	↓	▬	▬	▬	▬	▬	▬
	M. communis	▬	↓	↓	▬	▬	↓	▬	▬	▬
	O. vulgare	↓	↓	↓	↓	▬	↓	▬	▬	▬
	S. officinalis	↓	↓	▬	▬	▬	▬	▬	▬	↓
	S. rosmarinus	↓	↓	▬	▬	▬	↓	↓	▬	↓

		Ampicillin	Aztreonam	Ciprofloxacin	Ceftriaxone	Erytromycin	Gentamicin	Meropenem	Streptomycin	Tetracycline
E. faecalis	*C. nepeta*	↓	▬	↓	▬	▬	↓	▬	↓	▬
	C. bergamia	▬	▬	↓	▬	▬	▬	▬	▬	▬
	C. limon	↓	▬	↓	▬	▬	▬	▬	▬	↓
	C. reticulata	↓	▬	↓	↓	▬	▬	▬	▬	▬
	F. vulgare	↓	▬	↓	↓	▬	↓	▬	↓	↓
	L. nobilis	↓	▬	↓	↓	▬	↓	▬	▬	▬
	M. communis	↓	▬	↓	↓	▬	↓	▬	▬	▬
	O. vulgare	↓	▬	↓	↓	↓	↓	▬	▬	↓
	S. officinalis	↓	▬	↓	↓	▬	▬	▬	▬	▬
	S. rosmarinus	↓	▬	↓	↓	▬	↓	▬	↓	↓

Figure S1. Schematic representation of the changes induced in the MIC values of antibiotics by their combinations with EOs. The arrows indicate a decrease of MIC while the rectangles represent no change.

Table S1. Chemical components of the Essential Oils analysed in the study (presence ≥ 1%).

Essential Oil	Family	Chemical Composition	%	Retention Index
Clinopodium nepeta	*Lamiaceae*	piperitone oxide	34.28	16.14
		piperitenone oxide	18.23	19.72
		(+)-limonene	15.8	8.49
		(+)-pulegone	13.75	15.6
		menthone	8.32	12.77
		isolegylacetate	3.64	17.92
		1-terpine-4-ol	1.4	13.56
		(+)-neomenthol	1.37	13.25
		β-pinene	1.22	6.92
Citrus bergamia	*Rutaceae*	(+)-limonene	15.89	34.28
		lynalyl acetate	10.78	11.54
		(+)-linalool	9.38	6.79
		α -terpinene	8.44	38.88
		β-pinene	6.87	5.49
		α -pinene	5.64	1.22
Citrus limon (L.)	*Rutaceae*	(+)-limonene	14.13	3.01
		α -terpinene	13.59	1.26
		β-pinene	10.28	1.67
		α -terpineol	9.36	11.91
		α -terpinolene	8.42	74.41
		1-Terpine-4-ol	6.84	4.34
Citrus reticulata	*Rutaceae*	(+)-sabinene	12.6	1.44
		(+)-linalool	10.76	18.27
		α -phellandrene	10.27	1.37
		β -cis-ocimene	9.36	1.14
		(+)-limonene	8.96	6.45
		β -myrcene	8.42	5.04
		β -pinene	7.76	6.54
		α -pinene	7.16	2.37
		β -citronellal	6.86	2.35
		α -terpinolene	6.72	50.91
		α -terpinene	5.63	1.93
Foeniculum vulgare subsp. *piperitum*	*Apiaceae*	estragole	17.21	14.54
		α -pinene	14.19	45.33
		anethal	10.41	11.24
		fenchone	8.42	8.49
		α -limonene	7.72	2.51
		α -phellandrene	7.18	1.05
		β -pinene	6.86	1.65
		β -myrcene	5.63	14.71
Laurus nobilis L.	*Lauraceae*	eucalyptol	21.07	1.51

		(+)- sabinene	19.18	6.48
		(+)-linalool	13.6	1.29
		terpinyl acetate	10.79	7.38
		α -pinene	8.56	56.61
		methyleugenol	6.73	15.74
		1-terpine-4-ol	5.64	5.65
Myrtus communis L.	*Myrtaceae*	eucalyptol	20.27	1.88
		(-)-myrtenylacetate	18.43	17.04
		α -pinene	15.92	3.88
		(+)-limonene	14.16	2.1
		(+)-linalool	10.82	10.43
		lynalyl acetate	10.33	1.1
		geraniol acetate	9.44	1.09
		α -terpineol	9.04	1.58
		β -ocimene	8.59	33.04
		α -phellandrene	8.49	10.81
		o-cymene	8.35	1.41
		terpinolene	7.79	1.41
		terpinene	5.69	12.33
Origanum vulgare L. subsp. *viridulum*	*Lamiaceae*	p-thymol	20.48	4.88
		terpinene	16.77	47.31
		p-cymene	16.42	3.52
		β -caryophyllene	8.63	18.52
		β -myrcene	7.61	11.78
		carvacrol	7.36	3.18
		terpinolene	6.55	3.76
		α -thujene (origanene)	5.15	1.23
		α -pinene	4.96	2.73
Salvia officinalis L.	*Lamiaceae*	eucalyptol	22.6	5.54
		(-)-α -thujone	21.5	2.85
		β -pinene	12.45	9.59
		(-)-camphor	11.44	4.35
		α -humulene	11.07	24.14
		(-)-β -thujone	8.58	23.7
		α -pinene	7.23	2.26
		(-)-β - caryophyllene	6.9	15.1
		β -myrcene	6.75	1.13
		C camphene	6.12	1.88
		(+)- sabinene	5.67	3.99
Salvia rosmarinus	*Lamiaceae*	eucalyptol	21.52	1.17
		α -pinene	13.31	2.28
		β -pinene	12.45	3.66
		camphene	8.56	49.29

		(-)-camphor	7.21	1.79
		isoborneol	6.89	9.26
		β -myrcene	6.1	6.7
		(-)-β -caryophyllene	5.66	22.84

Table S2. Scalar concentrations of each antibiotic (µg/mL) tested in combination with the scalar dilutions of EOs (mg/mL) for *E. coli*, *P. aeruginosa* and *E. faecalis*.

	Ampicillin	Aztreonam	Ciprofloxacin	Ceftriaxone	Erytromicin	Gentamicin	Meropenem	Streptomycin	Tetracycline	EOs
E. coli	200.00	0.36	2.40	0.60	1200.00	50.00	0.20	50.00	20.00	5.00
	100.00	0.18	1.20	0.30	600.00	25.00	0.10	25.00	10.00	2.50
	50.00	0.09	0.60	0.15	300.00	12.50	0.05	12.50	5.00	1.25
	25.00	0.05	0.30	0.08	150.00	6.25	0.03	6.25	2.50	0.63
	12.50	0.02	0.15	0.04	75.00	3.13	0.01	3.13	1.25	0.31
	6.25	0.01	0.08	0.02	37.50	1.56	0.01	1.56	0.63	0.16
	3.13	0.01	0.04	0.01	18.75	0.78	0.00	0.78	0.31	0.08
	1.56	0.00	0.02	0.00	9.38	0.39	0.00	0.39	0.16	0.04
P. aeruginosa	4000.00	80.00	0.80	200.00	1260.00	3.20	0.24	8.00	800.00	5.00
	2000.00	40.00	0.40	100.00	630.00	1.60	0.12	4.00	400.00	2.50
	1000.00	20.00	0.20	50.00	315.00	0.80	0.06	2.00	200.00	1.25
	500.00	10.00	0.10	25.00	157.50	0.40	0.03	1.00	100.00	0.63
	250.00	5.00	0.05	12.50	78.75	0.20	0.02	0.50	50.00	0.31
	125.00	2.50	0.03	6.25	39.38	0.10	0.01	0.25	25.00	0.16
	62.50	1.25	0.01	3.13	19.69	0.05	0.00	0.13	12.50	0.08
	31.25	0.63	0.01	1.56	9.84	0.03	0.00	0.06	6.25	0.04
E. faecalis	20.00	4000.00	16.00	25.00	2000.00	100.00	200.00	1000.00	12.00	5.00
	10.00	2000.00	8.00	12.50	1000.00	50.00	100.00	500.00	6.00	2.50
	5.00	1000.00	4.00	6.25	500.00	25.00	50.00	250.00	3.00	1.25
	2.50	500.00	2.00	3.13	250.00	12.50	25.00	125.00	1.50	0.63
	1.25	250.00	1.00	1.56	125.00	6.25	12.50	62.50	0.75	0.31
	0.63	125.00	0.50	0.78	62.50	3.13	6.25	31.25	0.38	0.16
	0.31	62.50	0.25	0.39	31.25	1.56	3.13	15.63	0.19	0.08
	0.16	31.25	0.13	0.20	15.63	0.78	1.56	7.81	0.09	0.04
